# Differential Effects of Typical Korean Versus American-Style Diets on Gut Microbial Composition and Metabolic Profile in Healthy Overweight Koreans: A Randomized Crossover Trial

**DOI:** 10.3390/nu11102450

**Published:** 2019-10-14

**Authors:** Ji-Hee Shin, Sunhee Jung, Seong-Ah Kim, Min-Sook Kang, Min-Sun Kim, Hyojee Joung, Geum-Sook Hwang, Dong-Mi Shin

**Affiliations:** 1 Department of Food and Nutrition, College of Human Ecology, Seoul National University, 1 Gwanak-ro, Gwanak-gu, Seoul 08826, Korea; sjihee@snu.ac.kr; 2 Research Group of Healthcare, Research division of Food Functionality, Korea Food Research Institute, 245 Nongsaengmyeong-ro, Iseo-myeon, Wanju-gun, Jeollabuk-do 55365, Korea; 3 Integrated Metabolomics Research Group, Western Seoul Center, Korea Basic Science Institute, 150 Bugahyeon-ro, Seodaemun-gu, Seoul 03759, Koreamskim@kfri.re.kr (M.-S.K.); 4 Department of Chemistry, Sungkyunkwan University, 2066 Seobu-ro, Jangan-gu, Suwon-si, Gyeonggi-do 16419, Korea; 5 Department of Public Health, Graduate School of Public Health, Seoul National University, 1 Gwanak-ro, Gwanak-gu, Seoul 08826, Korea; ksacute@snu.ac.kr (S.-A.K.); hjjoung@snu.ac.kr (H.J.); 6 Institute of Health and Environment, Seoul National University, 1 Gwanak-ro, Gwanak-gu, Seoul 08826, Korea; 7 Department of Agro-food Resources, National Institute of Agricultural Sciences, Rural Development Administration, 166 Nongsaengmyeong-ro, Iseo-myeon, Wanju-gun, Jeollabuk-do 55365, Korea; mskang0803@korea.kr; 8 Department of Chemistry and Nano Science, Ewha Womans University, 52 Ewhayeodae-gil, Seodaemun-gu, Seoul 03760, Korea; 9 Research Institution of Human Ecology, Seoul National University, 1 Gwanak-ro, Gwanak-gu, Seoul 08826, Korea

**Keywords:** Korean diet, American diet, gut microbiota, metabolomics, enterotype, randomized crossover trial

## Abstract

The Westernized diet has been associated with the pathogenesis of metabolic diseases, whereas a Korean diet has been reported to exert beneficial effects on health in several studies. However, the effects of Western and Korean diets on the gut microbiome and host metabolome are unclear. To examine the diet-specific effects on microbiome and metabolome, we conducted a randomized crossover clinical trial of typical Korean diet (TKD), typical American diet (TAD), and recommended American diet (RAD). The trial involved a 4-week consumption of an experimental diet followed by a 2-week interval before diet crossover. 16S rRNA sequencing analysis identified 16, 10, and 14 differential bacteria genera specific to TKD, RAD, and TAD, respectively. The Firmucutes-Bacteroidetes ratio was increased by TKD. Nuclear magnetic resonance metabolome profiling revealed that TKD enriched branched chain amino acid metabolism, whereas ketone body metabolism was evident in RAD and TAD. Microbiome and metabolome responses to the experimental diets varied with individual enterotypes. These findings provide evidence that the gut microbiome and host metabolome rapidly respond to different cultural diets. The findings will inform clarification of the diet-related communication networks of the gut microbiome and host metabolome in humans.

## 1. Introduction

Numerous studies over the past several decades have clarified the close relationship between some diets and the prevalence of various chronic diseases such as cardiovascular disease, obesity, type 2 diabetes, and dyslipidemia [1]. In addition to the roles that nutrients and food components play in disease prevention and health maintenance, the dietary pattern has been increasingly recognized as being important, as human meals consist of a combination of various foods rather than a single food component or nutrient [2,3,4]. In this regard, dietary guidelines have been established in several countries to provide a standard for healthy meals [5]. Despite the differences in culture and food availability in many countries, it is typically considered that a healthy dietary pattern is predominantly characterized by a high intake of vegetables, fruits, and whole-grain foods, and a low intake of saturated fats, added sugars, and sodium [6]. 

The human body harbors an estimated 100 trillion commensal microbes, which dwell in sites including the skin, vagina, and the oral and gastrointestinal (GI) tracts [7]. The gut microbiota influences the uptake and metabolism of dietary components, such as fermentation of dietary fiber, synthesis of specific vitamins, and transformation of cholesterol [8]. Moreover, the gut microbiota influences defenses against pathogen colonization and maintains the homeostasis of the immune system [9]. In this regard, the composition of the gut microbiota is closely related to human health and a microbial imbalance may be associated with the pathology of several diseases including inflammatory bowel disease, obesity, and non-alcoholic fatty liver disease [10]. The composition of the gut microbiota depends on intrinsic factors such as sex, ethnicity, and age [11,12], and extrinsic factors that include diet, hygiene, antibiotic usage, and mode of delivery [13]. Among the environmental factors, diet is one of the major players in shaping gut microbial communities [14]. 

The intestinal microbiota features substantial inter-individual and intra-individual variations. Therefore, stratification of bacterial communities based on differences in the enrichment of the microbial taxa can be a way to simplify the complexity of the gut microbiota [15]. Enterotype describes the class of human gut microbiome based on its bacteriological ecosystem. Initially, three classes of human enterotypes were described [16]. Recent considerations include the influence of enterotypes on the susceptibility of the disease risk factors [17,18,19] and specific food ingredients [20]. A cross-sectional analysis of dietary information and the gut microbiome in humans showed that the *Prevotella*-dominant enterotype is associated with high intake of fiber, carbohydrate, and simple sugars, whereas *Bacteroides*-dominant enterotype is associated with the high intake of animal fat and protein [21]. Although robust evidence indicates that diet pattern is closely associated with gut microbial enterotypes, scant data are available from well-designed clinical trials concerning the effect of dietary intervention on individual enterotypes.

Multi-omics technologies that include genomics, transcriptomics, proteomics, and metabolomics are leading us toward a new approach for nutrition research [22]. In particular, metabolomics reflects the most current biological status of an individual by comprehensively analyzing and quantifying metabolites in samples, such as biofluids and feces [23,24]. This information could help clarify the molecular mechanisms of the effects of diet on health and disease as the closest link to phenotype [25,26]. Diet provides the substrates to the gut microbiota for the production of numerous metabolites, which are absorbed by the host and can alter host metabolism [27]. Analysis of the metabolites generated in the gut microbiome provides a snapshot of the relationship between the microbiome and human health. Additionally, metabolomics approaches can be applied to investigate the endogenous metabolome derived from food catabolism or nutrition in the host metabolism [28].

Over the past few decades, South Korea has experienced a rapid transition from the traditional Korean diet to a Westernized diet [29]. Simultaneously, chronic metabolic diseases have increased dramatically in prevalence [30,31,32]. Epidemiological studies have provided evidence of a relationship between Westernized diet and disease prevalence in Koreans. However, no systematic study has compared Korean diet to Western diets in terms of the modulation of the gut microbiome and metabolome profiles, including the underlying molecular mechanisms. Furthermore, only a few studies have addressed the interaction of diet and gut microbiome in humans, and the studies were restricted to European diets [33,34,35] or Western diets [36,37,38].

The current study aimed to examine the effects of the standard Korean diet and the Western diet on metabolic and gut microbiota profiles in healthy Korean adults. Previous findings of the substantial inter-individual variations in the human microbiome and metabolome [39,40] prompted us to examine the microbiome and metabolome changes occurring in the same individuals in response to different dietary patterns. The dietary patterns tested included the typical Korean diet (TKD), typical Western diet (typical American diet, TAD), and recommended healthy American diet (RAD). The study hypothesis was that the different types of diet might affect the communication between the microbiome and the host metabolome in an enterotype-specific manner. To test our hypothesis, we designed a randomized crossover clinical trial and determined the changes of gut microbiome and metabolome before and after 4-week consumption of the three different diets in 61 participants. These results provide insights into an individual’s response to different types of diet and will inform the development of strategies to control metabolic diseases and promote public health.

## 2. Materials and Methods

### 2.1. Participants and Study Design

Individuals were recruited in the area neighboring Seoul National University in Seoul, Korea. They were screened for their eligibility to participate in the study. Inclusion criteria were adult men and women aged from 25 to 65 years and body mass index (BMI) ≥23 kg/m^2^. Exclusion criteria included the following: (1) regular tobacco smoking; (2) patients with type 1 and/or 2 diabetes; (3) history of eating disorders or other abnormal eating habits; (4) history of major surgery of the GI tract and uncontrolled GI disorders or disease; (5) consumption of commercial pro-, pre-, and antibiotics within 6 months of the start of the study; (6) any regular intake of medication or undergoing treatment with cholesterol or blood pressure, kidney, liver, gastrointestinal, or endocrine disorders; and (7) body weight loss >10% or self-reported alcohol abuse within 12 months prior to screening. Enrolled participants were asked to report their usual diet for 3 days before the study started. The habitual diet compositions were computed using CAN-Pro 5.0 (Computer Aided Nutritional Analysis Program, the Korean Nutrition Society, Seoul, Korea) software. The present study was a randomized, three-period, crossover trial. Participants were supposed to consume each of TKD, RAD, and TAD for 4 weeks, separated by 2-week intervals for washout. They were randomly assigned to six groups according to the sequence of the diet change after stratification by sex and BMI. All meals including snacks were provided daily. Specifically, participants took breakfast at the research center from Monday through Friday; lunch, dinner, snacks, and weekend meals were packed for takeout.

In the beginning and at the end of each diet intervention, fecal, urine, and fasting blood serum were collected (Supplementary Figure S1). The study was conducted from November 2015 through January 2017. Informed consent was obtained from all participants. This study was approved by Seoul National University Institutional Review Board (IRB No. 1506/002-014) and registered with the Clinical Research Information Service (CRIS, Cheongju, Korea) of the Korea (registration No. KCT0002437).

### 2.2. Study Diets

The three trial diets were as detailed in the previous study [41]. Briefly, the meal plan for the TKD, RAD, and TAD diets were designed based on the Korean Food Guide of the Dietary Reference Intakes for Koreans [42,43], Sample Menus for a 2000 Calorie Food Pattern from the 2010 Dietary Guidelines for Americans developed by the United States Department of Agriculture [44], and What We Eat in America dietary survey from NHANES 2001–2004 [45], respectively. The TKD, RAD, and TAD were designed to match the macronutrients composition. The TKD featured 60%–65% of energy from carbohydrates, 15% from protein, and 20%–25% from fat. The RAD featured 55% of energy from carbohydrates, 15% from protein, and 30% from fat. The TAD featured 50% of energy from carbohydrates, 15% from protein, and 35% from fat. The sodium was provided 2.7, 1.7, and 2.7 g/day/2000 kcal in TKD, RAD, and TAD, respectively. Each participants’ diet composition was computed using the CAN-Pro 5.0 software and was adjusted to ensure that body weight was maintained throughout the end of each diet intervention periods. To monitor the maintenance of initial body weight (change of <5% of initial body weight), each participant’s body weight was measured daily for 5 days (Monday-Friday) every week.

### 2.3. 16S rRNA Gene Sequencing and Microbiome Data Analysis

Total bacterial DNA was isolated from fecal samples using the QIAamp^®^ DNA stool Mini Kit (QIAGEN, Hilden, Germany) according to the kit protocol for pathogen detection with a few modifications. Bacterial 16S rRNA gene amplicons were amplified with universal primers, which anneal to the hypervariable region V1–V2 of the bacterial 16S rRNA gene. Libraries were then sequenced by an Ion S5™ XL platform (Thermo Fisher Scientific, Waltham, MA) according to the manufacturer’s instructions. The sequenced 16S reads were analyzed by using QIIME 1.9.1. All of detailed methods have been described previously [46]. The methods of enterotype stratification previously described and available in https://enterotype.embl.de/enterotypes.html [16]. 

### 2.4. Metabolomic Analysis Based on Nuclear Magnetic Resonance (NMR)

For NMR analysis of serum samples, 200 μL of serum was mixed with 400 μL of saline solution (0.9% sodium chloride in deuterium oxide, D_2_O) and transferred to 5-mm NMR tubes. ^1^H NMR spectra of serum were acquired on an Advance III HD 800 MHz NMR spectrometer (Bruker BioSpin, Ettlingen, Germany) with a Bruker 5 mm CPTCI Z-GRD probe. The water-suppressed CPMG spin-echo pulse sequence (RD-90°-(τ-180°- τ) n-ACQ) was used. For all spectra of each serum, 128 transients were acquired with 64 k data points, spectral width of 16,025.641 Hz, relaxation delay of 4 s, and acquisition time of 2.045 s. For urine samples, 180 μL of urine was mixed with 360 μL of 100 mM sodium phosphate buffered D_2_O (pH 7.0), and 60 μL of 1 mM 3-(trimethylsilyl) propionic-2,2,3,3-d4 acid (TSP-d4) dissolved in D2O. The mixture was transferred to 5-mm NMR tubes. Nuclear overhauser enhancement spectroscopy (NOESY)-presat, RD-180°-mixing-90°-Acq, pulse sequences were used. For all spectra of each urine, 128 transients were acquired with 64 k data points, a spectral width of 16,393.443 Hz, and the relaxation delay of 4 s, and acquisition time of 1.998 s. 

All acquired ^1^H NMR spectra were phased, and baseline corrected using TopSpin 3.1 software (Bruker BioSpin, Rheinstetten, Germany) and Chenomx NMR Suite Version 7.1 (Chenomx, Edmonton, AB, Canada). The chemical shift was referenced to the signal of formate at 8.45 ppm in serum spectra and TSP in the urine spectra. Resonance assignments for serum and urine metabolites were accomplished using the 800 MHz library of Chenomx NMR Suite Version 7.1 (Chenomx, Edmonton, AB, Canada), two-dimensional NMR spectra, and spiking experiments. The synthetic electronic reference signal (ERETIC, electronic reference to access in vivo concentration) was used instead of DSS in the serum sample [47,48]. Targeted metabolic profiling of serum and urine were performed using NMR Suite Version 7.1 (Chenomx, Edmonton, AB, Canada) by integrating peak areas of metabolites compared with the areas of the known reference signal peak. Representative 800 MHz ^1^H NMR spectra of sera and urine are shown in Supplementary Figures S2 and S3, respectively.

### 2.5. Statistical Analyses

Multivariate statistical analyses were performed with unit variance scale by using SIMCA-P+ software, version 12.0 (Umetrics, Umea, Sweden). The Statistical Package for Social Sciences software, version 15.0 (SPSS Inc., Chicago, IL, USA), R studio, version 1.1.453, and GraphPad Prism, version 7.0a (GraphPad Software, Inc., La Jolla, CA, USA) were used to assess statistical significance using the Wilcoxon Signed-Rank test, Mann-Whitney U-test, Spearman’s correlation analysis. 

## 3. Results

### 3.1. Crossover Intervention

A total of 132 individuals were recruited and 61 eligible participants entered the randomized crossover trial (Supplementary Figure S1). Individuals began by consuming an experimental diet—the TKD, TAD, or RAD—that included three meals and one snack per day. Each diet was continued for 4 weeks, followed by a 2-week intervening period before beginning another diet. During the 2-week washout period, the participants resumed their usual diet. This procedure was repeated until all three experimental diets were completed. Seven participants dropped out during the trial, so data analysis included 54 paired (before/after) samples of TKD, 54 paired samples in the TAD period, and 53 paired samples in the RAD period (one RAD paired sample was omitted due to an early termination) (Supplementary Figure S1). Baseline characteristics of the study participants are summarized in Supplementary Table S1.

### 3.2. Changes of Alpha- and Beta-Diversity after Diet Intervention

**Figure 1:**
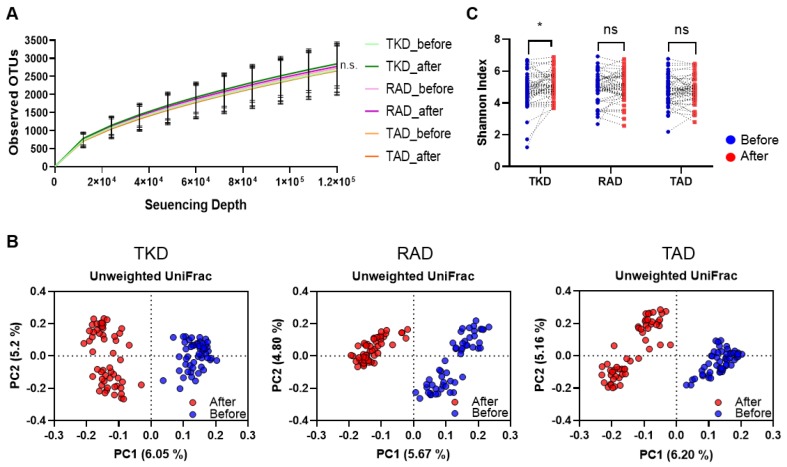
Effects of three different dietary patterns on the diversity of gut microbiota. 16S rRNA gene sequence analyses of gut microbiome were performed before and after typical Korean diet (TKD), recommended American diet (RAD), and typical American diet (TAD) interventions in crossover design. (**A**) Rarefaction curves of observed operational taxonomic units (OTUs). (**B**) Two-dimensional principle coordinate analysis based on the unweighted UniFrac distance of all sequenced samples before and after TKD, RAD, and TAD intervention. Each circle represents the profile of gut microbiome from each individual. Axes represent percentage of data explained by each coordinate dimension. (**C**) Changes in the diversity of intestinal bacterial communities determined by Shannon index. Paired comparison of before and after a dietary intervention was carried out and the significance was tested by Wilcoxon Signed-Rank test (* *p* < 0.05; ns, not significant).

To evaluate the effect of experimental diets on gut microbiota profiles within an individual, we performed 16S rRNA sequencing analysis of bacterial genomic DNAs from stool samples that were collected before and after each dietary intervention. After preprocessing of the bacterial sequences for quality control, as described in the Materials and Methods, a total of 88,176,057 raw reads and an average of 272,990.9 ± 75,289.6 reads per sample were obtained. Rarefaction curves of the number of operational taxonomic units (OTUs) indicated that the sequencing depth was sufficient for the further analysis of commensal bacterial community profiles (Figure 1A). Next, unweighted UniFrac distance-based principal coordinates analysis (PCoA) was performed to examine beta-diversity. The PCoA plot clearly distinguished the post-treatment microbial community profiles from pre-treatment samples in the TKD, RAD, and TAD samples (Figure 1B). To find out if the TKD, RAD, or TAD intervention led to a change in diversity of bacterial communities within an individual, the Shannon index for alpha-diversity was determined in each fecal sample. In a paired comparison before and after intervention, TKD promoted the diversity, whereas RAD and TAD did not (*p* = 0.0286; Figure 1C). The collective data indicated that, regardless of the type of diet, the experimental diet did induce changes in the bacterial communities from the state that existed when the usual diet was being consumed. In addition, these results indicated that consumption of TKD might promote gut microbial diversity.

### 3.3. Differential Effect of TKD, RAD, and TAD on Gut Microbiota Profiles

**Figure 2:**
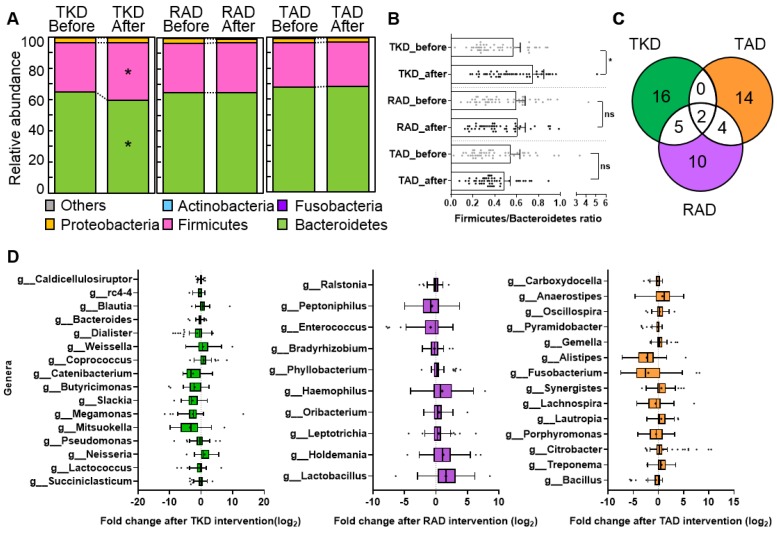
Differential effects of Korean diet and American diets on profiles of intestinal bacterial communities. (**A**) Relative proportions of gut microbial phyla before and after three diet intervention. (**B**) The ratio of Firmicutes to Bacteroidetes between before and after each diet intervention. (**C**) Venn diagram showing numbers of genera differentially changed in response to TKD, RAD, and TKD intervention (*p* < 0.05; Wilcoxon Signed-Rank test). (**D**) List of significant genera in comparison of before and after dietary intervention. Box plots of relative abundance show the fold change of after vs. before intervention in log_2_ scale. Green, purple, and orange color indicates TKD, RAD, and TAD, respectively.

To further investigate the specific bacterial taxa that were significantly affected by the experimental diets, we compared the composition of gut microbiota before and after TKD, RAD, and TAD intervention. At the phylum level, Bacteroidetes and Firmicutes were the predominant bacterial phyla in all subjects, in accordance with other reports [49,50]. After TKD consumption, Firmicutes was significantly increased (*p* = 0.016) and Bacteroidetes was significantly decreased (*p* = 0.006) compared to before TKD (Figure 2A). Consequently, a significant increase in the ratio of Firmicutes/Bacteroidetes was found in the comparison of before vs. after TKD intervention (Figure 2B, *p* = 0.0042). The levels of those two major bacteria were not changed by either RAD or TAD.

At the genus level, we identified 23, 21, and 20 differential genera in TKD, RAD, and TAD, respectively (*p* < 0.05). Venn diagram analysis determined the numbers of genera unique to TKD, TAD, and RAD; seven of 23 genera in TKD were overlapped with RAD, whereas only two genera in TAD overlapped (Figure 2C). *Caldicellulosiruptor, Blautia, Weissella, Coprococcus,* and *Neisseria* were significantly increased after TKD intervention, whereas *rc4-4, Bacteroides, Dialister, Catenibacterium, Butyricimonas, Slackia, Megamonas, Mitsuokella, Pseudomonas, Lactococcus*, and *Succiniclasticum* were markedly decreased in their relative abundance (Figure 2D). The RAD intervention increased *Phyllobacterium, Haemophilus, Oribacterium, Leptotrichia, Holdemania,* and *Lactobacillus*, but decreased *Ralstonia, Peptoniphilus, Enterococcus*, and *Bradyrhizobium*. After TAD intervention, there was a significant increase in *Anaerostipes, Oscillospira, Gemella, Synergistes, Lautropia, Citrobacter,* and *Treponema*. Furthermore, TAD intake resulted in a substantial reduction in *Carboxydocella, Pyramidobacter, Allistipes, Fusobacterium, Lachnospira, Porphyromonas,* and *Bacillus* (Figure 2D). These observations that diets dramatically changed gut microbial communities in a dietary pattern specific manner prompted us to further examine the host responses to diet by performing global profiling analysis of metabolome in the host.

### 3.4. Differential Effects of TKD, RAD, and TAD on Metabolic Profiles and Pathways

**Figure 3:**
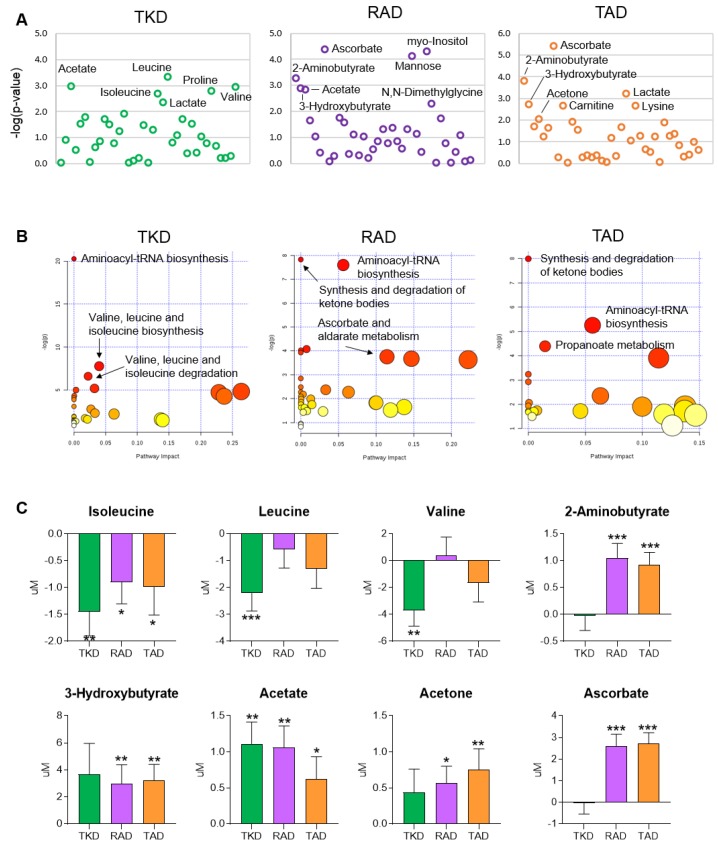
Global profiling analysis of serum metabolites. Metabolomic profiles of serum samples were determined by ^1^H NMR. (**A**) Significantly changed metabolites between before and after dietary intervention using Wilcoxon Signed-Rank test. Metabolites with raw *p* < 0.01 (−log_10_ of the *p*-value > 2) are labeled. (**B**) Significant metabolic pathways affected by diet intervention using Metaboanalyst pathway analysis. The color and size of the circles reflect the *p*-values and pathway impact values, respectively. (**C**) Alterations in the concentrations of metabolites. The vertical axis shows the alternation of metabolite concentration, which is the delta value between concentration of sample from before each diet and sample from after each diet (C_After_ minus C_Before_). *p*-value of significant differences between the levels before and after each dietary pattern were determined from the Wilcoxon Signed-Rank test. *, **, and *** indicate *p* < 0.05, *p* < 0.01, and *p* < 0.001, respectively.

To investigate the changes in metabolism after the consumption of each diet, we performed metabolic profiling using NMR spectroscopy and identified a total of 37 and 48 metabolites in serum and urine, respectively. Univariate statistical analysis was used to identify significantly changed metabolites affected by each dietary intervention. The differences in metabolite levels between before and after TKD, RAD, and TAD intervention in serum and urine are summarized in Supplementary Tables S2 and S3, respectively. Serum metabolites with significant changes between before and after dietary intervention using Wilcoxon Signed-Rank are shown in Figure 3A. Serum metabolite levels of acetate, isoleucine, leucine, lactate, proline, and valine were significantly altered after TKD intervention. Levels of 2-aminobutyrate, 3-hydroxybutyrate, acetate, ascorbate, mannose, myo-inositol, and N, N-dimethylglycine were changed after RAD intervention. TAD intervention changed the levels of 2-aminobutyrate, 3-hydroxybutyrate, acetone, ascorbate, carnitine, lactate, and lysine.

To further investigate the effect of each diet on metabolism, pathway enrichment analysis of the metabolomics data was carried out. The metabolic pathways affected by each dietary intervention in serum profile are shown in Figure 3B. The findings indicated that the enriched metabolic pathways were different among the three diets. Notably, the pathways related to valine, leucine, and isoleucine metabolism were highly enriched only after TKD intervention. In the RAD and TAD interventions, the predominant metabolic enrichment was the synthesis and degradation of ketone bodies. The findings indicated that host metabolism could be changed by the composition of the diet. Consistently, the serum levels of branched chain amino acids (BCAAs) were significantly decreased only after TKD intervention. Serum levels of 2-aminobutyrate, 3-hydroxybutyrate, acetate, and acetone (i.e., ketone bodies) were mostly elevated in RAD and TAD (Figure 3C). 

In urine samples, the levels of glycolate and taurine were significantly changed after all dietary interventions (Supplementary Figure S4A). Urinary metabolite levels of 3-hydroxy-3-methylglutarate (3-HMGA), citrate, dimethylamine, hippurate, and homovanillate were significantly altered after RAD intervention, whereas levels of 1-methylnicotinamide, carnitine, and pyruvate were changed after TAD intervention. Pathway analysis of urinary metabolic profiles revealed enriched taurine and hypotaurine metabolism in all dietary interventions. Glyoxylate and dicarboxylate metabolism and the citrate cycle (TCA cycle) were highly enriched after RAD and TAD (Supplementary Figure S4B). The level of citrate was increased in RAD and TAD, whereas the level of pyruvate was decreased in TKD and TAD. In addition, the levels of glycolate and taurine were altered in all diet interventions (Supplementary Figure S4C).

### 3.5. Influence of Enterotype on Responses of Microbial Communities and Host Metabolites to the Different Types of Diets

**Figure 4:**
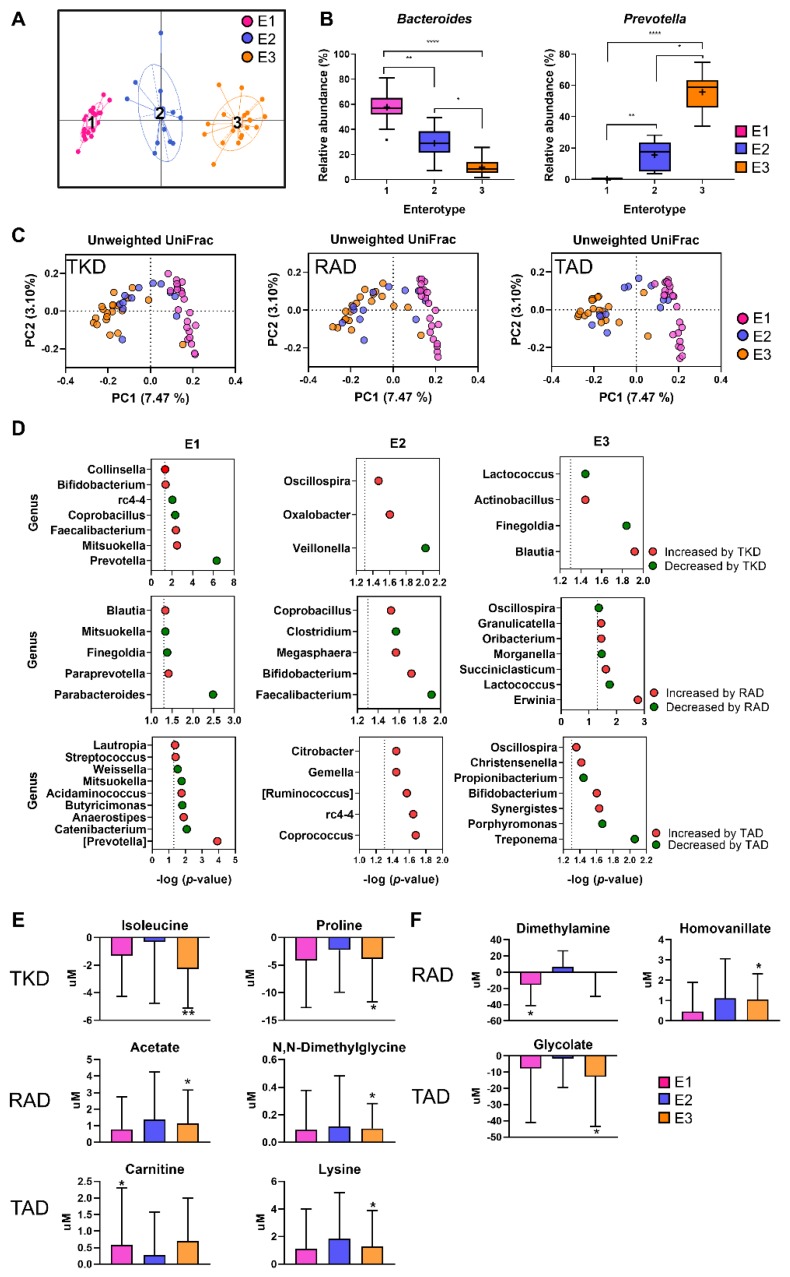
Effect of enterotypes on the microbial and host responses to experimental diets. (**A**) Classification of enterotypes by principal coordinates analysis of 54 individuals based on the composition of the genus level relative abundance profiles. (**B**) Tukey box and whiskers plots of the relative abundance of *Bacteroides* and *Prevotella* in each enterotype. Asterisk represents statistical significance (* *p* < 0.05, ** *p* < 0.01, **** *p* < 0.0001; Kruskal–Wallis followed by a Dunn’s multiple comparisons test). (**C**) PCoA plot of the unweighted UniFarc distances of gut microbial composition from three enterotypes after TKD, RAD, and TAD intervention. (**D**) Changes in the relative abundance of genera from before to after intervention were evaluated by Wilcoxon Signed-Rank test in each enterotype. Enterotype-specific increased and decreased bacterial genera colored red and green, respectively. The x-axis indicates the −log_10_ of the *p*-value of each genus from Wilcoxon Signed-Rank test between before and after each dietary intervention in each enterotype. Metabolites changes in serum (**E**) and urine (**F**) according to enterotype after each diet intervention. The y-axis indicates changes of concentrations of the metabolites (C_After_ minus C_Before_). Significant differences between the levels before and after dietary intervention were determined by the Wilcoxon Signed-Rank test. * and ** indicate *p* < 0.05 and *p* < 0.01, respectively. Enterotype 1 (E1), enterotype 2 (E2), and enterotype 3 (E3) are shown in pink, blue, and orange, respectively.

The human gut microbiome is based on its bacteriological ecosystem, and comprises enterotypes [16]. Next, we addressed whether changes in gut bacterial composition and metabolites in the three diets could be attributed to individual enterotypes. We stratified the gut microbiota of a set of participants using Partitioning Around Medoids, and Jensen-Shannon Divergence distance clustering algorithm [16]. The baseline compositions of gut microbial genera from the 54 participants separated into three distinct clusters of Enterotype 1 (E1), Enterotype 2 (E2), and Enterotype 3 (E3) (Figure 4A). The optimal number of clusters was determined by the Calinski-Harabasz index (Supplementary Figure S5A). Of the 54 participants, 22 belonged to E1, 12 to E2, and 20 to the E3 enterotype. Kruskal–Wallis testing revealed that *Bacteroides* and *Prevotella* were the significant bacteria that distinguished enterotypes (Figure 4B). The percentage of *Bacteroides* was overrepresented in E1 individuals (97.74%), whereas *Prevotella* was overrepresented in E3 individuals (83.71%). The E2 group had a rather balanced ratio of *Bacteroides* (65.00%) and *Prevotella* (35.00%) (Supplementary Figure S5B). General characteristics at baseline were not significantly different among the three enterotype groups (*p* > 0.05) (Supplementary Table S4).

To test whether enterotypes harbor distinct microbial communities after dietary intervention, we compared the beta-diversity across three different gut enterotypes after TKD, RAD, and TAD intervention. PCoA revealed that the structure of the gut microbiota differed significantly among gut enterotype groups after each dietary intervention (Figure 4C). To further investigate whether bacterial taxa significantly differed among the three gut enterotypes following each dietary intervention, we compared the gut microbiota composition at the genus level in each enterotype after TKD, RAD, and TAD intervention. By comparing before and after each dietary intervention, we identified differentially changed genera in E1, E2, and E3 (Figure 4D). 

*Collinsella*, *Faecalibacterium*, and *Mitsuokella* were significantly increased in E1 after TKD intervention, whereas *Prevotella* was markedly decreased (all *p* < 0.05; Figure 4D). *Actinobacillus* and *Blautia* were significantly increased in E3 after TKD intervention, whereas *Lactococcus* was markedly decreased (all *p* < 0.05; Figure 4D). In the RAD intervention, there were five, five, and seven genera unique to E1, E2, and E3, respectively. In the TAD intervention, *Lautropia* and *Streptococcus* were significantly increased in E1, whereas *Butyricimonas* and *Catenibacterium* were markedly decreased (all *p* < 0.05; Figure 4D). *Oscillospira* and *Christensenella* were significantly increased in E3, whereas *Propionibacterium* was markedly decreased (all *p* < 0.05; Figure 4D). Interestingly, *Bifidobacterium*, which is a well-known probiotic with health benefits in humans, was increased in abundance in the gut by TKD in E1 type individuals, by RAD for E2 type individuals, and by TAD in E3 type individuals, implying that the responses of *Bifidobacterium* to each dietary pattern were not the same to all individuals and varied depending on the specific enterotype. Taken together, these observations imply that changes in intestinal microbial communities driven by dietary intervention might be dependent on the enterotype of the host. In addition, we compared the concentration of specific metabolites (listed in Figure 3A and Supplementary Figure S4A) in each enterotype before and after TKD, RAD, and TAD intervention. Six and three metabolites in serum and urine, respectively, showed different changes of concentration according to enterotypes (Figure 4E,F). Serum concentration of isoleucine was significantly decreased only in the E3 group after TKD intervention. After RAD intervention, concentration of serum acetate was significantly increased only in the E3 group. Serum carnitine level significantly increased only in the E1 group after TAD intervention. Concentration of urinary dimethylamine was significantly decreased only in the E1 group after TAD intervention. Although the changes in host metabolites are less dramatic than those in gut microbiome, our results suggest that the enterotypes could partially affect specific response of host metabolites to the dietary intervention.

## 4. Discussion

Growing attention is being paid to the role of diet in modulating the gut microbiome and consequently affecting various physiological processes involved in human health and disease [8]. To our best of knowledge, this is the first intervention study to examine the effects of different dietary patterns on the gut microbiome and host metabolome profiles within the same individuals, using Korean and American diets. The Korean diet is characterized by high levels of vegetable, whole grains, and low levels of animal-derived foods and saturated fat [51]. On the other hand, a typical American diet, also known as the Western diet, is characterized by high levels of processed meat, added sugars, and saturated fat and low intakes of vegetable, fiber, and fruits [52]. In this study, we also examined a healthy American diet to compare the diet to the typical American diet and/or Korean diets on the intestinal bacterial communities and host metabolome. The healthy American diet tested in this study was based on the 2010 Dietary Guideline for Americans provided by the United States Department of Agriculture, which recommends a diet that is high in fruit, vegetables, low-fat dairy, whole grains, and lean protein foods and low in saturated fatty acid, sodium, and refined sugar and grains [53,54]. Differential effects of those diets were examined in overweight individuals in this study. Compared to normal weight individuals, obese and overweight individuals are more prone to develop microbial dysbiosis and metabolic disturbances [55,56]; therefore, the unbalanced shape of intestinal bacterial communities and dysregulated metabolome profiles were targeted and studied in the present study. To determine the diet-specific effects on the gut microbiome, we used a randomized crossover design in this study, because substantial variation between individuals is a well-known obstacle to microbiome studies in humans. This design enabled us to compare the different effects of the three distinct diets in the same individuals. However, the crossover design can suffer from a disadvantage called the carry-over effect, in which the consequences of one intervention could be carried over from one intervention period to another [55]. Therefore, to avoid a potential carry-over effect, we inserted a 2-week washout phase between each dietary intervention period. This period of washout was long enough to return the gut microbiome that had been changed by the experimental diet to the state of baseline within an individual (data not shown). 

One of the interesting observations was that the TKD decreased the abundance of phylum Bacteroidetes and genus *Bacteroides* in the intestinal bacterial communities. This result might reflect the difference in the levels of carbohydrate and animal protein in the diet, because TKD contains high levels of both digestible and non-digestible carbohydrates, and low levels of animal protein compared to TAD and RAD. This finding is consistent with previous studies [57,58], which negatively linked the levels of *Bacteroides* to the consumption of monosaccharides. In addition, the prevalence of *Bacteroides* has been reported to increase after consumption of a high meat diet compared to a meat-free diet, even before the next generation sequencing era [59]. Another recent report demonstrated that an animal-based diet was associated with high levels of *Bacteroides* because the bacterium is a bile tolerant microorganism [60]. A great deal of attention has been paid to Firmicutes since the first reports that their high abundance is a representative feature of gut microbiota composition in obese individuals [61,62,63]. However, the association of Firmicutes with obesity is still controversial, since recent studies conducted in populations from Belgium [64], Spain [65], and Korea [66] failed to reproduce the findings. While some studies showed that high animal fat diet and protein diet attributed the increased abundance of Firmicutes [35,67,68], other studies reported that plant-based diet increased Firmicutes [60,69]. We observed an increase in abundance of Firmicutes by the TKD; this diet contains more plant-derived components and less animal components than TAD and RAD, which agrees with the role of Firmicutes in metabolizing dietary plant polysaccharides [60].

Another characteristic of the Korean diet is the greater consumption of fermented foods [70]. The abundance of *Weissella* was significantly increased by TKD. *Weissella* is a probiotic bacterium that is crucial in the fermentation of Kimchi, a traditional fermented vegetable dish in Korea [71]. Previous studies reported that *Weissella* spp. has potential probiotic effects, such as reducing cholesterol [72,73], antioxidant properties [74], and antimicrobial activity [75]. *Lactobacillus* is one of the best-known probiotic bacteria and a major constituent of the lactic acid bacteria group. *Lactobacillus* is present in fermented milk products like yogurt and cheese, as well as Kimchi [76,77]. Therefore, we speculated that the abundance of *Lactobacillus* would be increased by a Korean diet. Contrary to this, the abundance of *Lactobacillus* was not affected by TKD, but was increased after RAD intervention. The daily meal plan for RAD included yogurt containing *Lactobacillus acidophilus* LAFTI^®^ L10 probiotic strain. This result indicates that a healthy American diet might be effective in promoting *Lactobacillus* population in the gut. Epidemiological studies have provided evidence that Westernized diets are positively related to the prevalence of cardiovascular disease, with the Korean diet being negatively related to this prevalence [78,79]. In the current study, TKD increased the prevalence of *Coprococcus* in the intestinal bacterial communities. This result might partly explain the beneficial effect of the Korean diet, because this taxon produces short chain fatty acids and therefore promotes cardiovascular health [80]. After TAD intervention, we observed a significant decrease in the number of *Lachnospira*. Recent studies demonstrated the negative association between the abundance of *Lachnospira* and very low-density lipoprotein cholesterol level [81,82]. Additionally, the low abundance of *Lachnospira* has been linked to obesity, elevated BMI [83], and scant consumption of vegetables [34]. 

Enterotypes have been classified into *Prevotella*- and *Bacteroides*-dominant groups in previous studies [84,85] and are reportedly influenced mainly by long-term dietary habits, among other environmental factors [21]. Consistent with previous studies [21,84,85,86], the relative composition of these two taxa distinguished one enterotype from the others in the present study population, and the classification was maintained throughout the study. To our knowledge, the present study is the first to identify the enterotype-specific responses to diet, and we observed the responses to TKD, TAD, and RAD were not the same for all enterotypes. It was of special interest to determine the changes in well-known beneficial bacteria—*Collinsella*, *Bifidobacterium*, and *Faecalibacterium*—in each enterotype. *Collinsella* can produce butyrate [87], which is a short chain fatty acid (SCFA), as a major product of fermented non-digestible carbohydrates. *Bifidobacterium* and *Faecalibacterium* promote intestinal health related to the production of SCFAs and maintenance of intestinal homeostasis [88]. In subjects with *Bacteroides*-dominant enterotype (E1), *Collinsella*, *Bifidobacterium*, and *Faecalibacterium* were increased in their relative abundance in response to TKD, but not to RAD or TAD. On the contrary, for individuals who had a balanced composition in *Bacteroides* and *Prevotella* (E2), RAD increased *Bifidobacterium* and *Faecalibacterium* in the gut. Surprisingly, for those in group with the *Prevotella*-dominated enterotype (E3), the abundance of *Bifidobacterium* was increased by TAD. These results indicated that the responses to diet were affected by the individual’s enterotypes and suggest that enterotype might be a significant variable that contributes to, modulates, or confounds experimental outcomes in studies of diet and the gut microbiome. Although enterotyping is assumed to be useful in predicting responses to drugs and diets, to our knowledge no intervention accessing the applicability has been reported. The results of this study provide evidence that an individual’s gut enterotype might be one of the crucial factors to develop personalized nutrition strategies for human health and well-being. 

To identity metabolic patterns relating to three different diets, we carried out metabolic profiling analysis of serum and urine samples using NMR. BCAAs and ketone body metabolism displayed diet-specificity in endogenous metabolism. TKD significantly decreased the levels of isoleucine, leucine, and valine, indicating the changes in the metabolism of BCAAs. Additionally, in pathway analysis, which provides greater power to detect important metabolic pathway associations with diet [89], we observed that the metabolism of BCAAs was significantly modulated by diets. It has been consistently reported that circulating levels of BCAAs are high in obese individuals and are associated with worse metabolic disease, which results from inhibiting insulin signaling and impaired glucose metabolism [90,91,92]. A clinical nutrition study reported that BCAAs are essential in physiological regulation related to diet characteristics [93]. For example, it was reported that rye bread intake lowered serum levels of leucine and isoleucine in a randomized crossover trial, with greater beneficial effects on insulin responses than that resulting from wheat bread intake [94]. Elin et al. demonstrated that BCAAs are one of the plasma metabolites that connect gut microbiota profiles to metabolic syndrome traits in humans [95]. Thus, our findings indicate that BCAAs might be the key metabolites in the network composed of diet, gut microbiome, and host responses. 

Ketone body related metabolites, such as 2-hydroxybutyrate, 3-hydroxybutyrate, acetone, and 3-hydroxyisovalerate were increased after both TAD and RAD. In addition, pathway and correlation analysis of serum using NMR revealed that identified ketone body metabolism as an important and influential mechanism in TD and RAD interventions. Ketone bodies are generated by fatty acid oxidation, and are necessary for the conversion of dietary lipids. Surplus fatty acid availability enhances fatty acid oxidation to produce ATP instead of glucose oxidation [96]. TKD is characterized by the high consumption of carbohydrate consisting mainly of rice, and low consumption of fat [97]. The alteration of the levels of ketone bodies was caused by the higher intake of fat or fatty acid, and lower intake of the carbohydrate than the participates’ basal diet. Compared to the Korean diet, RAD and TAD are relatively high in fat and fatty acid, and relatively low in carbohydrate, leading to up-regulated fatty acid oxidation. Therefore, the alteration of ketone body metabolism in RAD and TAD could be explained by high fat consumption through diet shift in the Korean participants, which is consistent with a previous diet intervention study regarding fatty acid oxidation [98].

Our findings may help clarify the effects of diet on the alteration of circulating metabolites and gut microbial profiles. However, there were some limitations that need to be overcome in future studies. First, it is unclear whether the alteration of the gut microbiota community and metabolites drive changes in host characteristics. Second, because the study participants were obese or overweight (BMI ≥ 23 kg/m^2^), our findings may not be applicable to individuals whose BMI is <23 kg/m^2^. Third, although global profiling analysis of the metabolome and microbiome generated a great deal of dimensional profile data in a robust manner, the specific molecules or bacteria species might not have been determined due to technological limitations. Lastly, metabolome and microbiome profiles were closely related to ethnic groups and geographical locations where people reside. Future studies should involve various groups and locations.

In conclusion, to our knowledge, this study compared for the first time the differential roles that a Korean diet and two American diets, healthy and typical, played in modulating intestinal bacterial communities and host metabolite profiles in a crossover design. We demonstrated that 4-week consumption of each diet dramatically changed the gut microbiome structure and serum metabolome networks in a diet-specific manner. Differences in Korean diet vs. American diets was much more dramatic than any other comparisons of three different diets. Although we found the diversity of bacterial communities and ratio of Firmicutes to Bacteroidetes at the phylum level were changed only by the Korean diet, at the genus level the taxon matrix specific to Koreans, typical American, and healthy American diets were identified. Global metabolome profiling analysis and pathway analysis revealed the metabolism of BCAAs and ketone bodies as significant networks that distinguished Korean diet and both American diets. Additionally, we demonstrated that a response of gut microbiome-host interaction to a diet might vary based on the individual’s enterotype. These findings indicate that the gut microbiome and host metabolome respond rapidly to different cultural diets and provide information that will be helpful for constructing the communication networks of the gut microbiome and host metabolome with roles of diet in orchestrating the communication in humans, and developing strategies for personalized nutrition.

## Supplementary Materials

The following are available online at https://www.mdpi.com/2072-6643/11/10/2450/s1, Figure S1: Scheme of the study protocol with a randomized crossover trial, Figure S2: Representative 800 MHz ^1^H NMR spectra of serum Figure S3: Representative 800 MHz ^1^H NMR spectra of urine, Figure S4: Effects of dietary interventions urinary metabolites profiles, Figure S5: Clustering of gut microbiota from participants in baseline, Table S1: Baseline characteristics of the participants, Table S2: List of serum metabolites, Table S3: List of urine metabolites, Table S4: Baseline characteristics of the subgroups defined by enterotype.
