# Supplementary material for: Differential Effects of Typical Korean Versus American-Style Diets on Gut Microbial Composition and Metabolic Profile in Healthy Overweight Koreans: A Randomized Crossover Trial
Source: Nutrients. 2019 Oct 14;11(10):2450. doi: 10.3390/nu11102450 (PMC6835328; doi:10.3390/nu11102450)
Supplement: Supplementary file 1 [file nutrients-11-02450-s001.zip › Supplementary Figure S1.pdf]

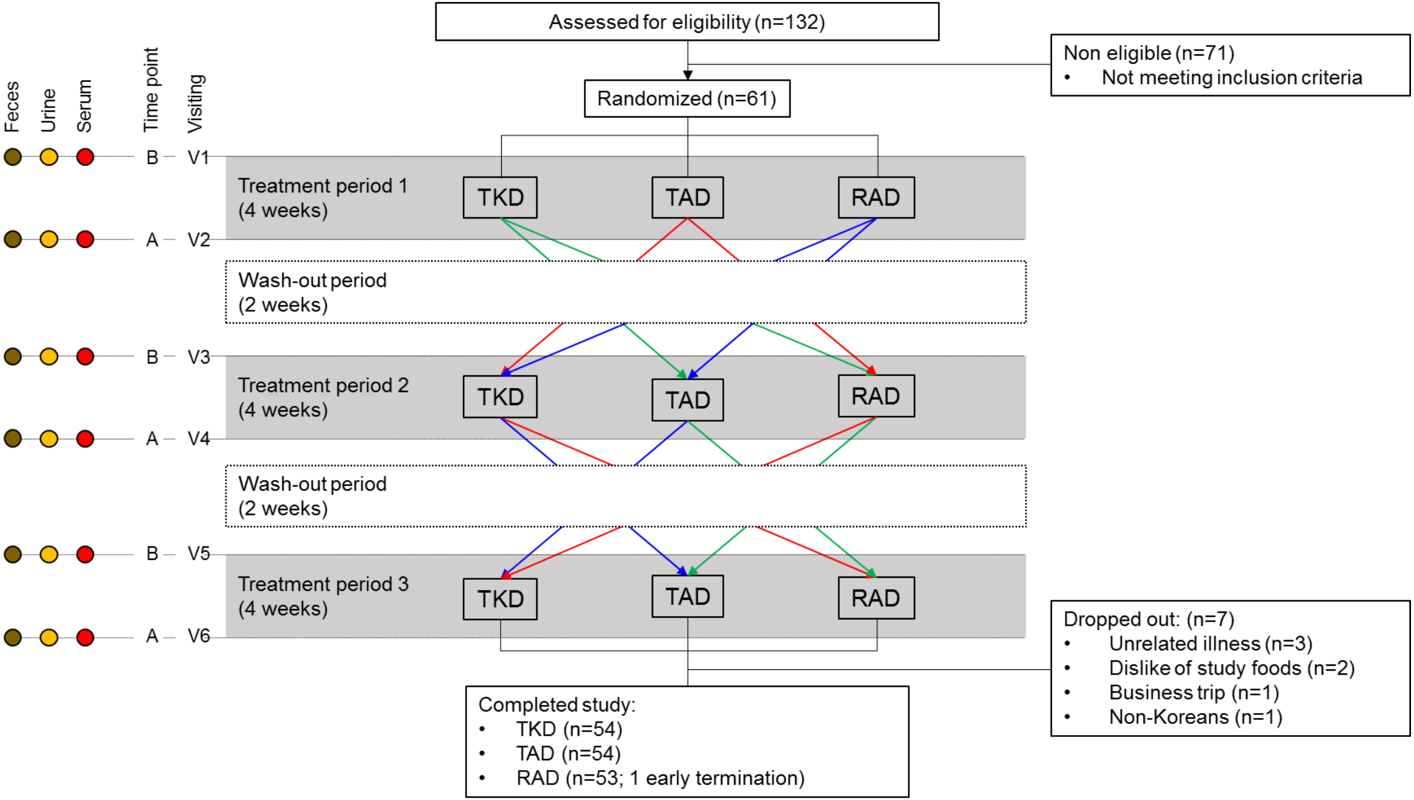

Supplementary Figure S1. Scheme of the study protocol with a randomized crossover trial. Abbreviations: TKD, typical Korean diet; RAD, recommended American diet; TAD, typical American diet.
