# Supplementary material for: Differential Effects of Typical Korean Versus American-Style Diets on Gut Microbial Composition and Metabolic Profile in Healthy Overweight Koreans: A Randomized Crossover Trial
Source: Nutrients. 2019 Oct 14;11(10):2450. doi: 10.3390/nu11102450 (PMC6835328; doi:10.3390/nu11102450)
Supplement: Supplementary file 1 [file nutrients-11-02450-s001.zip › Supplementary Figure S2.pdf]

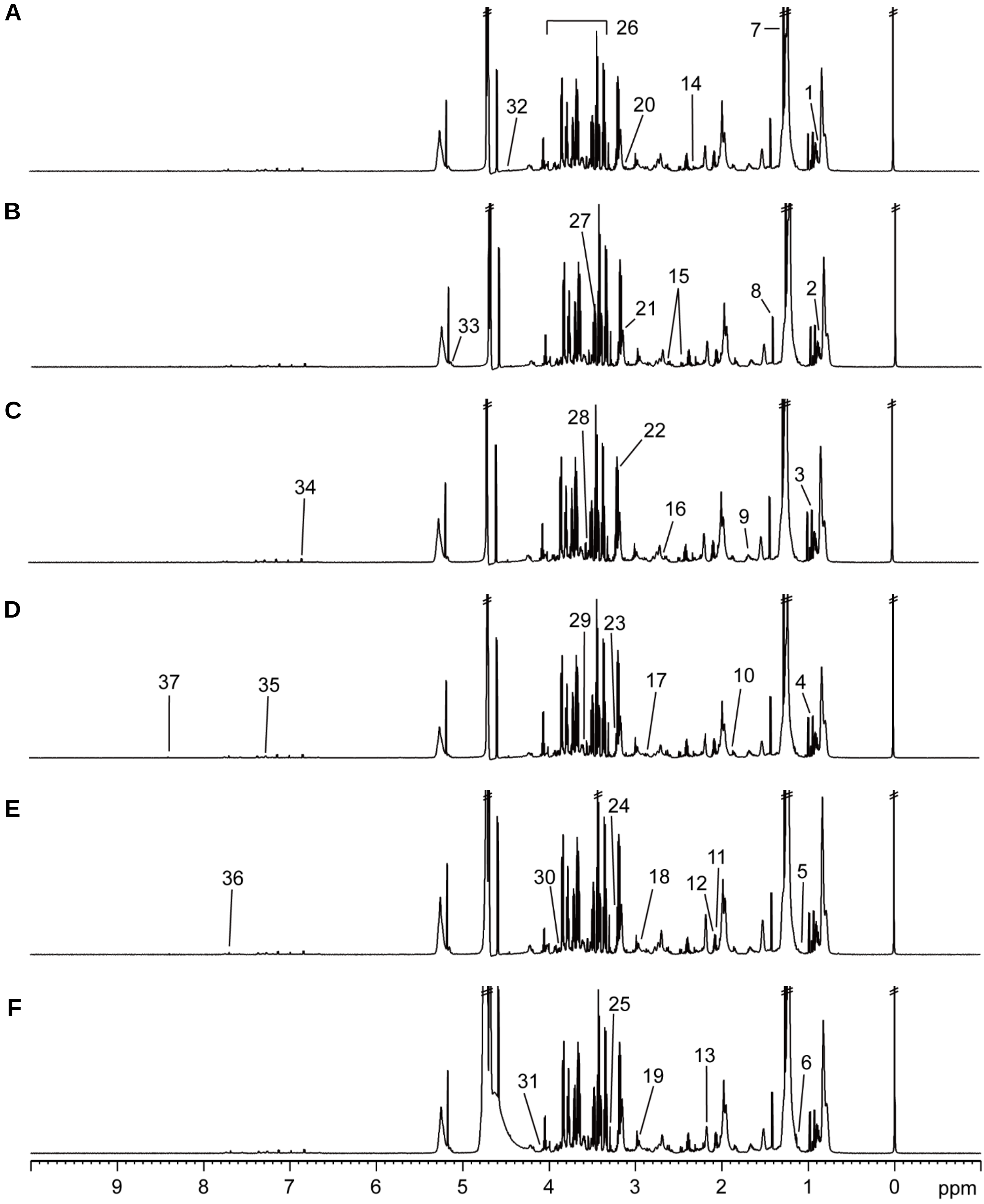

Supplementary Figure S2. Representative 800 MHz 1H NMR spectra of sera. The spectra of sera from participates before TKD (A); after TKD (B); before RAD (C); after RAD (D); before TAD (E); after TAD (F). Key: 1, Leucine; 2, 2-Aminobutyrate; 3, Valine; 4, Isoleucine; 5, Ethanol; 6, 3-Hydroxybutyrate; 7, Lactate; 8, Alanine; 9, Arginine; 10, Acetate; 11, Glutamine; 12, Methionine; 13, Acetone; 14, Pyruvate; 15, Citrate; 16, Dimethylamine; 17, N; N-Dimethylglycine; 18, Lysine; 19, Creatine; 20, O-Phosphocholine; 21, Choline; 22, Carnitine; 23, Trimethylamine N-oxide; 24, Betaine; 25, Methanol; 26, Glucose; 27, Glycine; 28, Threonine; 29, myo-Inositol; 30, Creatinine; 31, Proline; 32, Ascorbate; 33, Mannose; 34, Tyrosine; 35, Phenylalanine; 36, Histidine; 37, Formate.
