# Supplementary material for: Differential Effects of Typical Korean Versus American-Style Diets on Gut Microbial Composition and Metabolic Profile in Healthy Overweight Koreans: A Randomized Crossover Trial
Source: Nutrients. 2019 Oct 14;11(10):2450. doi: 10.3390/nu11102450 (PMC6835328; doi:10.3390/nu11102450)
Supplement: Supplementary file 1 [file nutrients-11-02450-s001.zip › Supplementary Figure S3.pdf]

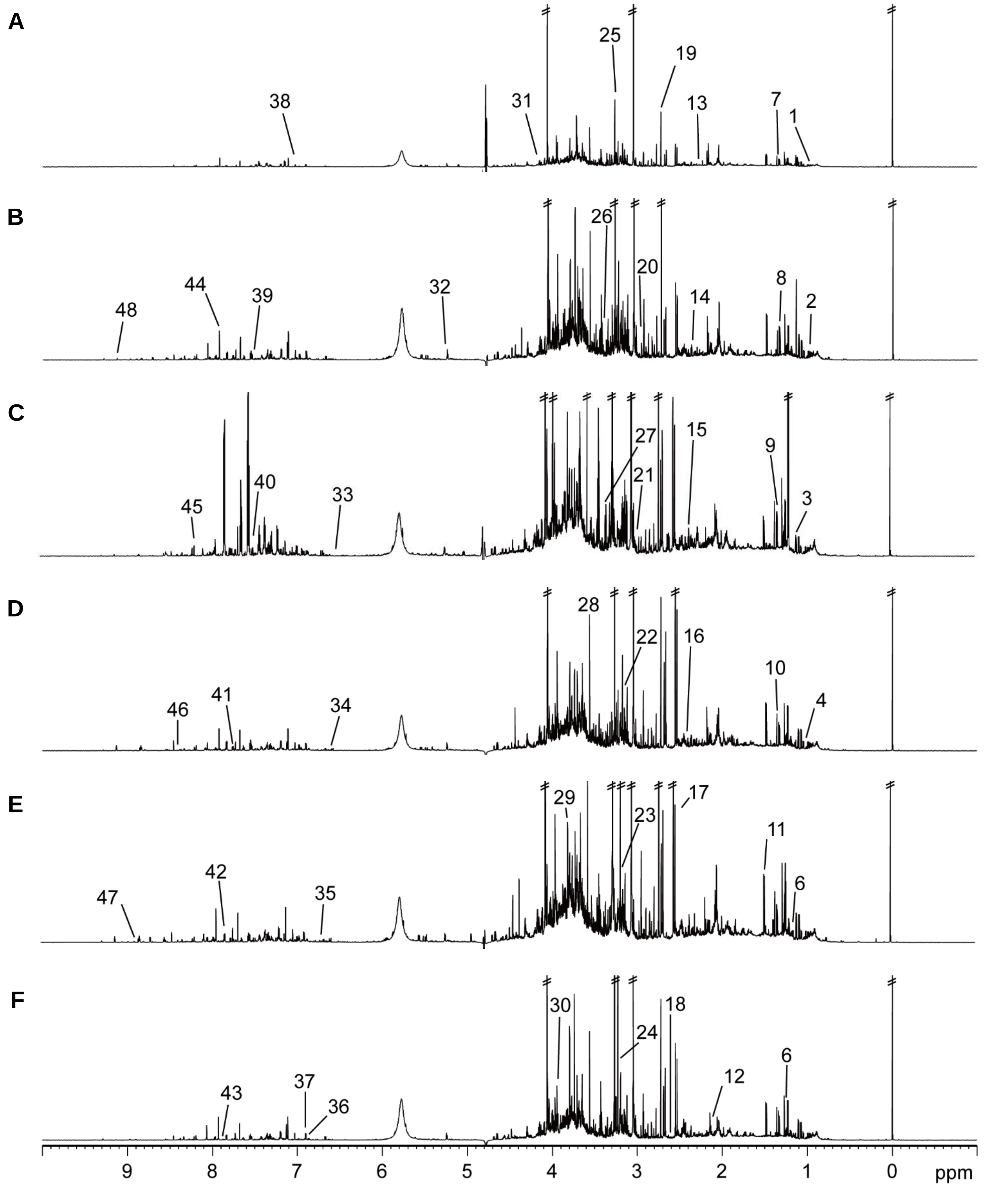

Supplementary Figure S3. Representative 800 MHz 1H NMR spectra of urine. The spectra of urine from participates before TKD (A); after TKD (B); before RAD (C); after RAD (D); before TAD (E); after TAD (F). Key: 1, 2-Hydroxybutyrate; 2, Leucine; 3, Valine; 4, Isoleucine; 5, Methylsuccinate; 6, 3-Hydroxyisovalerate; 7, 3-Hydroxy-3-methylglutarate; 8, Lactate; 9, Threonine; 10, 2-Hydroxyisobutyrate; 11, Alanine; 12, Methionine; 13, Acetone; 14, Pyruvate; 15, Succinate; 16, 2-Oxoglutarate; 17, Citrate; 18, 3-Aminoisobutyrate; 19, Dimethylamine; 20, N; N-Dimethylglycine; 21, Isocitrate; 22, cis-Aconitate; 23, Ethanolamine; 24, Carnitine; 25, Trimethylamine N-oxide; 26, Methanol; 27, Taurine; 28, Glycine; 29, Guanidoacetate; 30, Glycolate; 31, Gluconate; 32, Glucose; 33, Fumarate; 34, trans-Aconitate; 35, Homovanillate; 36, 4-Hydroxyphenylacetate; 37, Tyrosine; 38, τ-Methylhistidine (1-Methylhistidine); 39, Uracil; 40, 3-Indoxylsulfate; 41, Tryptophan; 42, Hippurate; 43, Xanthosine; 44, Histidine; 45, Hypoxanthine; 46, Formate; 47, 1-Methylnicotinamide; 48, Trigonelline.
