# Supplementary material for: Differential Effects of Typical Korean Versus American-Style Diets on Gut Microbial Composition and Metabolic Profile in Healthy Overweight Koreans: A Randomized Crossover Trial
Source: Nutrients. 2019 Oct 14;11(10):2450. doi: 10.3390/nu11102450 (PMC6835328; doi:10.3390/nu11102450)
Supplement: Supplementary file 1 [file nutrients-11-02450-s001.zip › Supplementary Figure S4.pdf]

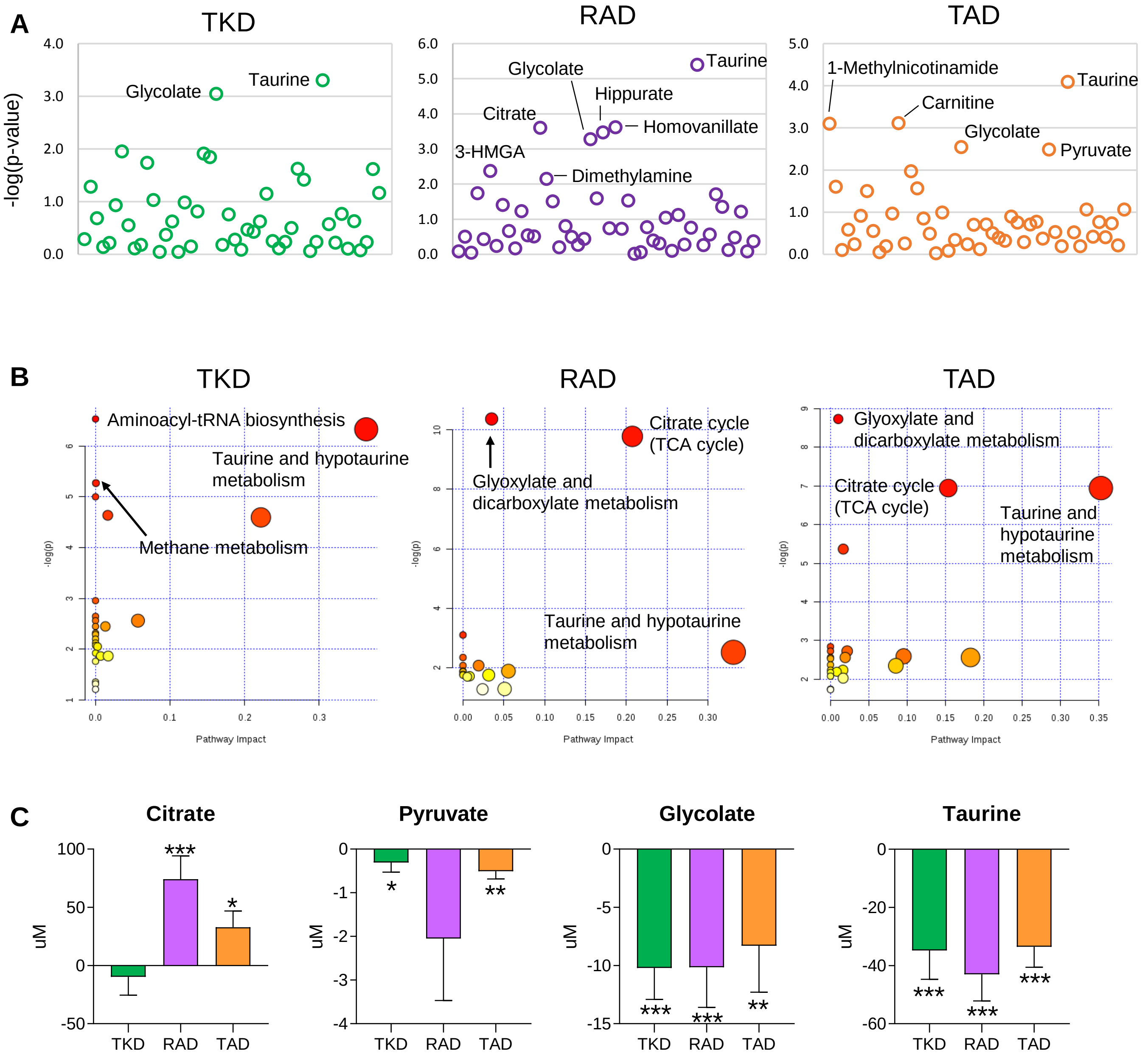

Supplementary Figure S4. Effects of dietary interventions urinary metabolites profiles (A) Plots of the -$\log_{10}$ of the P-value of each metabolites from Wilcoxon Signed-Rank test between before and after each diet intervention. Associations with raw P-value <0.01 (-$\log_{10}$ of the P-value >2) are labeled with metabolites name. 3-HMGA: 3-Hydroxy-3-methylglutarate. (B) Important metabolic pathways influenced by diet intervention using Metaboanalyst pathway analysis. The color and size of the circles reflect the p-values and pathway impact values, respectively. (C) Alterations in the concentrations of metabolites. The vertical axis shows the alternation of metabolite concentration, which is delta value between concentration of sample from before each diet and sample from after each diet (C_{After}-C_{Before}). P-value of significant differences between the levels before and after each dietary pattern were determined from the Wilcoxon signed-rank test. *, **, and *** indicate p < 0.05, p < 0.01, p < 0.001, respectively.
