# Supplementary material for: Differential Effects of Typical Korean Versus American-Style Diets on Gut Microbial Composition and Metabolic Profile in Healthy Overweight Koreans: A Randomized Crossover Trial
Source: Nutrients. 2019 Oct 14;11(10):2450. doi: 10.3390/nu11102450 (PMC6835328; doi:10.3390/nu11102450)
Supplement: Supplementary file 1 [file nutrients-11-02450-s001.zip › Supplementary Figure S5.pdf]

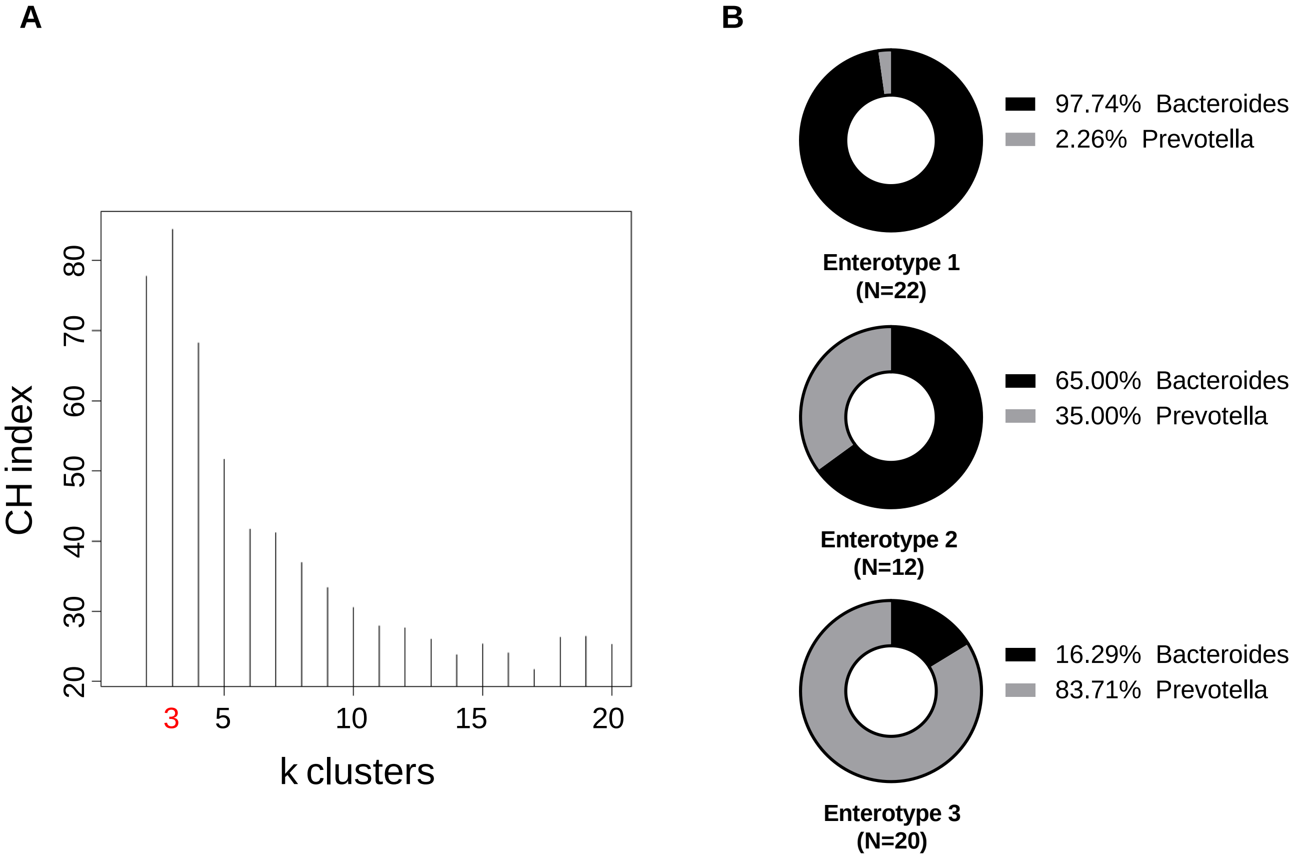

Supplementary Figure S5. Clustering of gut microbiota from participants in baseline (A) Optimal number of clusters calculated by Calinski-Harabasz (CH) index. (B) Relative proportions (percent) of *Bacteroides* and *Prevotella* in three different enterotype populations.
